# Utilizing retinal arteriole/venule ratio to estimate intracranial pressure

**DOI:** 10.1007/s00701-024-06343-0

**Published:** 2024-11-08

**Authors:** Mathias Just Nortvig, Mikkel Christian Schou Andersen, Niclas Lynge Eriksen, Jan Saip Aunan-Diop, Christian Bonde Pedersen, Frantz Rom Poulsen

**Affiliations:** 1 Department of Neurosurgery, Odense University Hospital, 5000 Odense, Denmark; 2 Clinical Institute and BRIDGE (Brain Research - Inter Disciplinary Guided Excellence), University of Southern, Southern Denmark, Denmark

**Keywords:** Intracranial Pressure (ICP), Fundoscopy, Retinal arteriole/venule ratio, Non-invasive monitoring, Trauma

## Abstract

**Purpose:**

Intracranial pressure (ICP) control is important to avoid secondary brain injury in patients with intracranial pathologies. Current methods for measuring ICP are invasive and carry risks of infection and hemorrhage. Previously we found correlation between ICP and the arteriole-venous ratio (A/V ratio) of retinal vessels in an outpatient setting. This study investigated the usability of fundoscopy for non-invasive ICP estimation with the addition of intraocular pressure (IOP) in patients in a neuro-intensive care unit (NICU).

**Methods:**

This single-center prospective cohort study was conducted at the NICU of Odense University Hospital from September 2020 to May 2021. Adult patients with a Glasgow Coma Score of 8 or less, who underwent invasive pressure neuromonitoring were included. Fundoscopy videos were captured daily and analyzed using deep learning algorithms. The A/V ratio was calculated and correlated with ICP. The data was analyzed using mixed-effect linear regression models.

**Results:**

Forty patients were enrolled. Fifteen were included in the final analysis. ICP ranged from -1 to 31 mmHg (mean: 10.9, SD: 5.7), and IOP ranged from 4 to 13 mmHg (mean: 7.4, SD: 2.1). The A/V ratio showed a significant negative correlation with ICP > 15 mmHg (regression slope: -0.0659, 95%-CI: [-0.0665;-0.0653], p < 0.001). No significant change in A/V ratio was observed for ICP ≤ 15 mmHg. A similar significant correlation was found for ICP > IOP (regression slope: -0.0055, 95%-CI: [-0.0062;-0.0048], p < 0.001). Taking the IOP into account did not improve the model. The sensitivity analysis showed a sensitivity of 80.08% and a specificity of 22.51%, with an AUC of 0.6389.

**Conclusion:**

In line with our previous work, non-invasive fundoscopy is a potential tool for detecting elevated ICP. However, challenges such as image quality and diagnostic specificity remains. Further research with larger, multi-center studies are needed to validate the utility. Standardization may enhance the technique's clinical applicability.

## Introduction

Intracranial pressure (ICP) control is important to avoid secondary brain injury in patients with intracranial pathologies [5, 7, 19]. Intracranial hypertension has been shown to increase mortality and morbidity in several conditions such as meningitis and severe head trauma [17, 19]. ICP exceeding 20 mmHg is usually considered elevated and measures should be taken to achieve ICP control [13].

The first ICP measure was performed by Queckenstedt in 1916 by a lumbar puncture [20]. However, it was Lundberg who performed the first catheterization to monitor and treat the ICP [20]. This method was accepted in the 1970s and has since 2007 been a key part of managing serve traumatic brain injury [4]. ICP is usually measured using invasive techniques such as intraparenchymal monitors or external ventricular drains (EVD). While these methods provide accurate readings, they require surgery and are thus associated with risks such as infection and hemorrhage [14, 16]. These procedures are typically performed in neurosurgical centers and are not readily available in hospitals without a neurosurgical unit or in prehospital settings. Because of this, there has been a growing interest in the development of reliable, non-invasive alternatives, which can be used without specialized training. This is particularly relevant in low-income countries and low dense populated areas with limited access to neuromonitoring, neuroimaging and treatments [6].

Several non-invasive ICP modalities have been investigated since the 1970s [11, 15]. However, none of these have yet been implemented clinically as continuous monitoring or screening tools. Retinal vessel diameter obtained through fundoscopy represents a novel development within the field of non-invasive ICP monitoring [2]. The basis for the modality is a measurement of the ratio between the diameter of the retinal arteriole and venule (A/V ratio). Theoretically, an increase in ICP compromise venous outflow, thus a distension of the venule and a lower A/V ratio may be observed [1, 9, 12, 18]. We demonstrated this phenomenon in 2020 [2], where we also showed a difference in the A/V ratio when ICP was above and below 15 mmHg. This study was, however, limited to patients with normal ICP. Furthermore, we suggested that the change in A/V ratio and correlation with ICP could be affected by the IOP, suggesting the combination of ocular hemodynamics and vascular compliance in relation to ICP.

By this study we utilize simple imaging techniques and advanced deep learning algorithms for retinal video analysis. This study aims to investigate the feasibility of using non-invasive fundoscopy to assess ICP by evaluating the correlation between the retinal A/V ratio and ICP in patients admitted to a neuro-intensive care unit (NICU). Moreover, the dynamic between ICP, IOP and A/V ratio will be investigated.

## Methods and materials

### Setting and study participation

This single-center, observational, prospective cohort study was performed in the Neuro Intensive Care Unit (NICU). The study was performed according to the Declaration of Helsinki and was approved by the Regional Committee on Health Research Ethics for Southern Denmark (S-20190107), the Danish Data Protection Agency (20/4618) and in agreement with current local and national guidelines and regulations. Participants were included from September 2020 to May 2021. Given the nature of the study, unconscious patients could be included initially without their personal consent in accordance with approved study protocol. Informed consent was obtained from next-of-kin either prior to or after the fundoscopy was performed. Patients who regained consciousness were asked for consent as well.

The inclusion criteria were patients aged ≥ 18 admitted to the NICU who underwent invasive pressure neuromonitoring with either external ventricular drainage (EVD) or an intraparenchymal pressure monitor (IPPM). All participants had a Glasgow Coma Score of 8 or less.

Retinal videos of both eyes were captured daily. The ICP was recorded with a standard webcam pointed at the patient monitor. The ICP was calculated by meaning ICP-values from 5 s before and after the image frame’s timestamp. ICP and A/V ratio were then correlated according to specific time stamps. No mydriatic drugs were used. The IOP was obtained after each video session. Clinical and demographic data were collected from the electronic patient records as shown in Table 1.

**Table 1:** Demographic data of the 15 patients included

Baseline characteristics, n = 15	
Gender (Male/Female)	4/11 (27/73)
Mean age (years), [SD]	62.13 [13.91]
Mean BMI (kg/m), [SD]	26.97 [4.27]
Diagnosis at admission	
Intracranial hemorrhage, n (%)	5 (33.3)
Subarachnoid hemorrhage, n (%)	5 (33.3)
Traumatic brain injury, n (%)	5 (33.3)

Fundoscopies were performed using the Epicam M camera (Epipole Ltd., Rosyth, UK), which is CE-marked and compliant with Quality Management Software with ISO 13485. The Epicam is a handheld monochromatic sensor image camera that produces serial still images (15 images/second) of 1280 × 1024 pixels and can be manually focused (± 15 diopters). The videos were obtained using designated Epicam software (Epicam viewer software 3.1.1., Epipole Ltd.). IOP was obtained by a CE-marked ic100 tonometer TA011 (Icare Finland Oy, Finland) that was approved by the Reduction of Hazardous Substances in Electrical and Electronic Equipment (RoHS) Directive 2011/65/EU and has an accuracy of ± 1.2 mmHg up to 20 mmHg and ± 2.2 mmHg for > 20 mmHg. Both tonometry and fundoscopy was performed by the same operator.

### Blinding

Fundoscopy and video analysis was done by separate individuals to ensure sufficient blinding and bias minimization. The fundoscopy operator matched each patient to the corresponding ICP acquired from the webcam data and performed statistics in collaboration with OPEN, SDU.

### Retinal video analysis

Retinal video processing and analysis was performed by an external company (Statumanu ICP ApS, Denmark). StatuManu was given access to pseudo anonymized data only. Retinal video analysis was performed using a deep learning model which was trained on more than 200,000 fundus and non-fundus images. It is partly based on an image classifier called Efficientnet [3]. The video recordings had a frame rate of 15 frames/second. The deep learning algorithm separated the videos into frames. It categorized each frame as ‘fundus’ or ‘no fundus’, based on the whether the optic disc and retinal vessels were present.

The optic disc was identified using ‘YOLOv7 object detection model’. Images without the optic disc present were discarded. Blood vessels in the remaining images were segmented using a deep learning vessel segmentation model. The highest quality image was chosen as a reference image, and its quality was assessed by an Edge Detection Filter (Tenengrad Gradient Magnitude) on a 1.5height/width crop around the optic disc. The remaining vessel segmentations were aligned to match the reference image.

The measurement points on each vessel were determined using a deep learning algorithm (Human Pose Estimation) [10]. This algorithm had been retrained on retinal fundus images with marked measurement points to identify measurement points instead of poses.

### Calculating vessel width and area

**Fig. 1:**
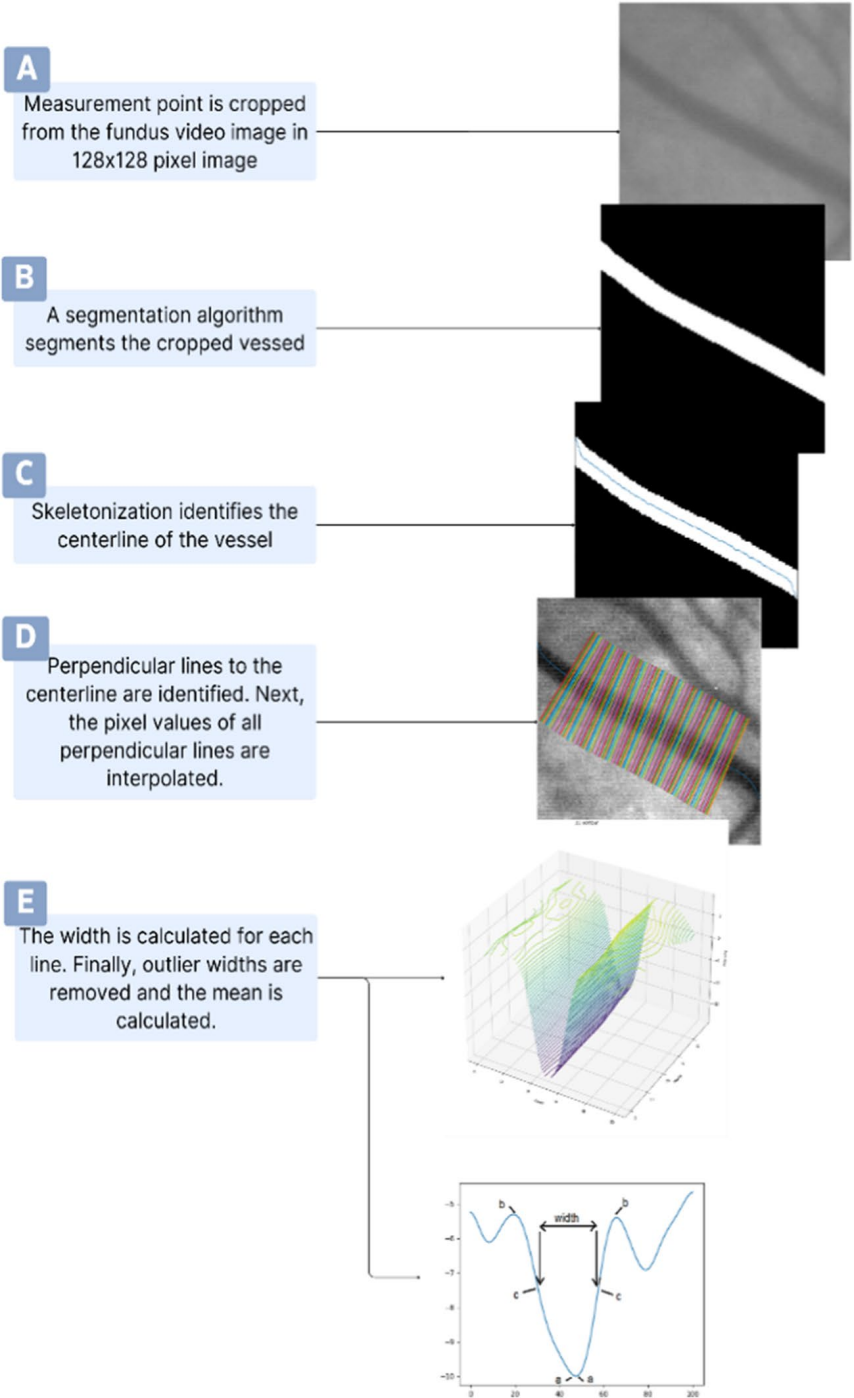
Step-by-step visual illustration of vessel width calculation. **A** For every frame vessel segmentation, each measurement point is cropped in a 128 × 128 pixel image. **B** The cropped vessel is segmented using a segmentation algorithm. **C** The centerline of the vessel is identified using skeletonization. **D** Perpendicular lines to the centerline are identified, and the pixel values of all perpendicular lines are interpolated. **E** The width of each line is calculated. Outlier widths are removed from the calculations, and the width mean is calculated

Using the defined measurement points, the widths of each vessel were calculated (Fig. 1). Using the calculated width, the area of a vessel at the point of a cross section could also be calculated:

$$Are{a}_{vessel}=\pi {\left(\frac{width}{2}\right)}^{2}$$

The ratio between the retinal artery and vein was calculated as the ratio between the areas of the two vessels:

$$A{V}_{area}=\left(\frac{{\left(\frac{arter{y}_{width}}{2}\right)}^{2}}{{\left(\frac{vei{n}_{width}}{2}\right)}^{2}}\right)$$

The A/V ratio was calculated for each measurement point and correlated with ICP according to time stamps.

### Statistical considerations

The data analysis was performed using Stata 17 (StataCorp LLC, College station, Texas, USA). Patients’ A/V ratio differs from one another as shown in our previous study [2] hence we used a mixed-effect linear regression model for the hierarchical structured data. The correlations between ICP, IOP and A/V ratio from the pooled data were assessed. Furthermore, the A/V ratio was correlated with ICP for ICP ≤ IOP and ICP > IOP for visualization. The two slopes were then compared using a Wald test to investigate whether ICP > IOP or ICP > 15 mmHg correlated better with the A/V ratio. This method has only been investigated in one study [2] and a power calculation could therefore not be performed. This study serves as a proof-of-concept study.

## Results

40 NICU patients were enrolled in the study. One patient withdrew consent and further 24 patients were excluded by the algorithm due to poor image quality. This left 15 patients included in the final analysis. Baseline characteristics are presented in Table 1. We analyzed a total of 7762 data points from 32 sessions with corresponding time matched ICP obtained from the NICU monitor device. IOP ranged from 4 to 13 mmHg (mean: 7.4, SD: 2.1), and ICP ranged from -1 to 31 mmHg (mean: 10.9, SD: 5.7). Six patients had ICP above 15 mmHg. There were no adverse events reported following the fundoscopy.

We grouped the patients according to reference ICP as either ICP ≤ 15 mmHg or ICP > 15 mmHg. We found a statistically significant coefficient (regression slope: -0.0659, 95%-CI: [-0.0665;-0.0653], p < 0.001) for the A/V ratio in ICP > 15 mmHg. In the group with ICP ≤ 15 mmHg there was no significant change in A/V ratio. (regression slope: -2.75 × 10^–14^, 95%-CI: [-0.0004;0.0004], p = 1.0) (Table 2).

**Table 2:** Regression analysis results comparing slopes by ICP and IOP thresholds

Group	Slope (Mean)	Std. Error	95% Confidence interval	p-value
ICP > 15 mmHg	-0.0659	0.0003	[-0.0665;-0.0653]	< 0.001
ICP ≤ 15 mmHg	-2.75 × 10^–14^	0.0004	[-0.0004–0.0004]	1.000
ICP > IOP	-0.0055	0.0003	[-0.0062;-0.0048]	< 0.001
ICP ≤ IOP	0.0022	0.0014	[-0.0006;0.0050]	0.131

Since IOP might affect vessel diameter due to the external pressure on the vessels, the analyses were repeated with IOP in focus. The correlation between ICP and A/V ratio given ICP > IOP showed a significant negative coefficient (regression slope: -0.0055, 95%-CI: [-0.0062;-0.0048], p < 0.001). However, for ICP ≤ IOP there was no significant coefficient (regression slope: 0.0022, 95%-CI: [-0.0006;0.0050], p = 0.131) (Table 2).

A Wald test was used to compare the slope coefficient of ICP > 15 mmHg with the slope coefficient of ICP > IOP. The difference between the slopes was found to be -0.0604 (SE: -0.0005, 95% CI: [-0.0613;-0.0595] (Table 3). The z-statistic was -130, with a corresponding p-value of < 0.0001. These results suggest that the slope of the relationship between the A/V ratio and ICP > 15 mmHg was significantly different from the slope with ICP > IOP. Specifically, the slope with ICP > 15 mmHg is significantly more negative compared to the slope with ICP > IOP. These findings indicate that the ICP has a greater impact on the A/V ratio than the IOP. Therefore, when the A/V ratio indicates increased ICP, the IOP is less relevant.

**Table 3:** Results of two-sample z test comparing slopes

Group	Mean (slope)	Std. Error	95% confidence interval
ICP > 15 mmHg	-0.065886	0.0003005	[-0.066475;-0.065297]
ICP > IOP	-0.0054981	0.0003393	[-0.0061631;-0.0048331]
Difference	-0.0603879	0.0004532	[-0.0612762;-0.0594996]

From the mixed-effect linear regression model, we found an interclass correlation coefficient (ICC) of 0.3895 (SE: 0.088, 95% CI: [0.236;0.568]), indicating that 39% of the variance in ICP measurements can be attributed to differences between patients. This is a moderate level of correlation, suggesting that there is a significant amount of variance explained by patient-specific factors. The model had an R-squared value of 0.5509, indicating, that 55.09% of the variance in ICP measurements can be explained by the predicted values from the model. The F-statistics for the model was 9520.03 (p < 0.001), demonstrating that the model is highly significant.

The modalities’ ability to differentiate between ICP ≤ 15 mmHg and ICP > 15 mmHg was assessed in a sensitivity analysis when not accounting for individual A/V ratios with an area under the curve (AUC) of 0.64. This analysis showed a sensitivity of 80.1%, a specificity of 22.5%, a positive predictive value of 75.7% and negative predictive value of 27.3%, with an A/V ratio cut-off point of 0.75.

## Discussion

This study assessed the potential of non-invasive fundoscopy to estimate ICP by assessing the correlation between the retinal A/V ratio, IOP and ICP. The findings indicate a significant change in A/V ratio with an increasing ICP, particularly > 15 mmHg or ICP > IOP. The slope with ICP > 15 mmHg was significantly more negative than the slope with ICP > IOP, and the stronger negative slope in the ICP > 15 mmHg indicates a more pronounced effect of the increasing ICP on the A/V ratio, whereas the influence of IOP appears to be less relevant. Therefore, it is evident that ICP has a greater impact on A/V ratio than IOP. Consequently, when the A/V ratio indicates increased ICP, the IOP is not of great importance. The significant negative correlation between the A/V ratio and ICP > 15 mmHg is the foundation of non-invasive fundoscopy’s potential of identifying patients with elevated ICP, since there is no significant change in A/V ratio for ICP ≤ 15 mmHg. Therefore, it is possible to detect relative changes in ICP if multiple measurement points are available on the same patient. The observed relationship supports the hypothesis that increased ICP affects retinal hemodynamics, leading to a lower A/V ratio due to venous distension and compromised venous drainage. This aligns with previous studies that have documented changes in retinal hemodynamics in response to intracranial pressure variations [2, 8, 12].

The study's sensitivity and specificity analysis of > / ≤ 15 mmHg revealed an area under the curve (AUC) of 0.64. Patients exhibit individual baseline A/V ratios but has comparable changes in A/V ratio correlating with changes in ICP [2, 8]. Despite this problematic physiological variance, the model still correctly identifies 80.1% of cases with ICP > 15 mmHg. However, only 22.5% of cases with ICP ≤ 15 is classified as negative, hence resulting in false positives. This low diagnostic specificity should in future studies be accounted for by improving image quality and investigating whether other variables could be implementing in the model. Further, it should be investigated in future studies if the AI-algorithm could provide retinal findings to enhance the diagnostic specificity and sensitivity.

Many non-invasive modalities have been investigated, but none of them have shown potential for measuring continues ICP, as the methods are unable to estimate the absolute ICP value [11]. Furthermore, there are no consensus on cut-off values, and sensitivity and specificity vary greatly between studies. This makes it difficult to compare A/V ratio with other non-invasive modalities.

This study has several limitations. Firstly, the small sample size, with only 15 patients included in the final analysis, limits the generalizability of the findings. The deep learning algorithm excluded 24 patients from the analysis due to poor image quality due to the widely used anesthetic fentanyl that induces miosis. The CE branded Epicam M camera is optimal for ≥ 4 mm pupils. Therefore, mydriatic drugs would greatly increase the image quality. The miosis and camera quality could also be reasons for the variance in A/V ratio, suggesting further studies also work on enhancing image quality. A higher quality camera able to perform fundoscopy on pupils ≥ 4 mm could limit the exclusion rate. Secondly, because of the risk of secondary brain injury, the patients were under tight ICP control in the NICU, thus only six patients had an ICP > 15 mmHg. The inaccessibility to high ICP in a human population suggests animal studies with fixed different ICP values should be performed to investigate correlation of high ICP > 25 mmHg.

In conclusion, this study demonstrates the potential of non-invasive fundoscopy as a tool for estimating ICP, corroborating previous findings from our group and enhancing understanding of ocular hemodynamics in relation to ICP. Despite challenges such as image quality and diagnostic specificity, our findings underscore the promise of fundoscopy in clinical applications, particularly in resource-limited settings where invasive monitoring is impractical or impossible. However, the technology is still not ready for implementation in clinical practice.

Future research should prioritize larger, multi-center studies to validate these findings across diverse patient populations and broader ranges of ICP. Additionally, exploring the integration of this non-invasive method into clinical practice and optimizing hardware are crucial steps forward. Animal studies investigating high ICP levels above 25 mmHg could provide further insight into the application of fundoscopy in neurocritical care.

## Data Availability

All obtained data are available upon reasonable requests. The authors Mathias Just Nortvig, Mikkel Schou Andersen or Frantz Rom Poulsen can be contacted on respectively mathias.just.nortvig@rsyd.dk, mikkel.c.schou.andersen@rsyd.dk or frantz.r.poulsen@rsyd.dk for data request.

## Abbreviations

AUC: Area under the curve
A/V ratio: Arteriole/Venule ratio
BMI: Body mass index
EVD: External ventricular drain
ICP: Intracranial pressure
IOP: Intraocular pressure
IPPM: Intraparenchymal pressure monitor
mmHg: Millimeters of mercury
NICU: Neuro intensive care unit
RoHS: Reduction of Hazardous Substances
SD: Standard deviation

## Declarations

### Clinical investigator

MD Asger Bjørnkær Nielsen assisted in obtaining data from included participant.

data request.
